# Epidemic characteristics of measles and efforts to control measles infections in Zhejiang Province, China

**DOI:** 10.4178/epih.e2024075

**Published:** 2024-09-11

**Authors:** Rui Yan, Mengya Yang, Hanqing He, Yan Feng, Yang Zhou, Xuewen Tang, Xuan Deng, Yao Zhu, Yuxia Du, Can Chen, Cao Kexin, Shigui Yang, RIDPHE Group

**Affiliations:** 1 Zhejiang Provincial Center for Disease Control and Prevention, Hangzhou, China; 2 Department of Epidemiology and Biostatistics, School of Public Health, Department of Emergency Medicine, Second Affiliated Hospital, Zhejiang University School of Medicine, Hangzhou, China; 3 Quzhou People’s Hospital, Zhejiang, China

**Keywords:** Measles, Epidemiological trends, Vaccines, China

## Abstract

**OBJECTIVES:**

Several countries have successfully eliminated measles, and China is making significant strides toward achieving this goal. This study focused on investigating the patterns of measles infections in Zhejiang Province, China, as well as control measures. The objective was to provide valuable insights that could contribute to the development of nationwide elimination strategies.

**METHODS:**

We analyzed measles surveillance data from 2005 to 2022 in Zhejiang Province. We utilized a joinpoint regression model to examine trends in measles. Additionally, we employed SaTScan version 9.5 to identify spatial-temporal clusters. Finally, we used an age-period-cohort model to assess the effects of age, period, and cohort.

**RESULTS:**

The age-standardized incidence rate (ASIR) of measles infection in Zhejiang Province from 2005 to 2022 was 5.24 per 100,000, showing a consistent and significant downward trend with an annual percentage change of -24.93% (p<0.05). After 2020, the ASIR for measles infection fell to below 0.1 per 100,000. The majority of measles cases occurred in individuals either without an immunization history or with an unknown immunization status, representing 41.06% and 41.40% of the cases from 2010 to 2022, respectively. According to data from the National Measles Surveillance System, the annual rate of discarded measles cases from 2009 to 2014, and the annual rate of discarded measles and rubella cases from 2015 to 2022, were both above 2 per 100,000, indicating the high sensitivity of the measles surveillance system.

**CONCLUSIONS:**

The significant reduction in measles incidence from 2005 to 2022 demonstrates substantial progress in Zhejiang Province towards the elimination of measles.

## GRAPHICAL ABSTRACT

[Fig f6-epih-46-e2024075]

## Key Message

Measles incidence in Zhejiang Province has been declining since 2008, with a particularly notable decrease from 2020 to 2022. Adults remain the primary population susceptible to measles in Zhejiang Province. Therefore, it is essential to enhance prevention and control measures for measles, especially among individuals who have not received the MCV immunization. The reduced incidence of measles during the COVID-19 pandemic presents Zhejiang Province with a unique opportunity to leverage the lowest recorded measles incidence in its history and accelerate efforts in measles elimination.

## INTRODUCTION

Measles, a highly contagious disease caused by the measles virus, is characterized by symptoms including fever and rash [1]. Although vaccination has significantly reduced global measles mortality, with an 83% decrease from 2000 to 2021, the disease continues to be prevalent in many developing countries, especially in regions of Africa and Asia [2].

Zhejiang, located on the east coast of China, has a population of over 59.98 million and spans an area of approximately 101,000 km^2^. The measles vaccine (MV) was first introduced in Zhejiang in 1967, with routine immunization established in 1978. This included a 2-dose schedule, with the first dose at 8 months and the second at 7 years, starting in 1986 [3]. In 2005, the immunization program was enhanced by adjusting the timing of the second dose from 7 years to 18-24 months. In 2008, China expanded the Expanded Program on Immunization to include the rubella-containing vaccine. Consequently, the measles and rubella vaccine (MR) was administered for the first dose at 8 months, and the measles-mumps-rubella vaccine (MMR) for the second dose at 18-24 months. In 2011, routine MR vaccination was introduced for 9th-grade students in Zhejiang Province. By 2019, the Zhejiang Provincial Government further improved the program by transitioning from MR to MMR for both 8-month-old infants and 9th-grade students. As a result, Zhejiang Province now implements a 3-dose MMR policy at 8-months, 18-24 months, and 13-14 years.

In 2010, the Western Pacific Regional Office of the World Health Organization (WHO) organized a global consultation that certified the feasibility of eradicating measles [4]. By 2020, the Global Vaccine Action Plan for 2012-2020 had set targets to eliminate measles in 5 WHO regions. By September 2013, all 6 WHO regions had adopted goals to eliminate measles by 2020 or earlier [5]. By the end of 2021, 76 countries were verified to have successfully eliminated measles [6]. The Region of the Americas was declared to have achieved measles elimination in 2000, a status officially confirmed in 2002, although it lost this status in 2018 [7]. Measles continues to be endemic in China, with ongoing efforts aimed at achieving elimination.

Measles cases in China have been declining since 2014, with the country making significant strides toward eliminating the disease [8]. The remarkable reduction in measles cases began in 2020, coinciding with the implementation of non-pharmaceutical interventions (NPIs) during the coronavirus disease 2019 (COVID19) pandemic [9]. Monitoring programs to eliminate measles requires the analysis of surveillance data and performance indicators. This study provides an epidemiological overview using data from the case-based measles surveillance system in Zhejiang Province, spanning from 2005 to 2022. Additionally, we will explore the implications of our findings and outline the necessary steps to sustain the decreasing trend of measles in Zhejiang.

## MATERIALS AND METHODS

### Surveillance system for measles

Since 2004, suspected cases of measles, which is a notifiable infectious disease, have been required to be reported to the National Notifiable Diseases Reporting System (NNDRS) within 24 hours of detection. The information collected for each patient includes their name, age, sex, residence, date of onset, and occupation. In addition to the NNDRS, the National Measles Surveillance System (MSS) was active from 2003 to 2009. In 2009, these two surveillance systems were merged, facilitating the online reporting of additional details such as vaccination status, clinical symptoms, and potential sources of infection for each patient. Following the 2009 revision of the National Measles Surveillance Program [10], the measles surveillance system’s sensitivity, timeliness, and specificity were enhanced. The MSS played a crucial role in improving the evaluation indicators of the surveillance system. The data on measles cases and deaths in Zhejiang used in this study were obtained from the NNDRS and MSS submissions. The annual incidence and morbidity rates (per 100,000 population) were calculated using the Zhejiang provincial census data, as reported in the statistical yearbook of Zhejiang Province. Measles incidence data for China from 2005 to 2022 were downloaded from the WHO website [11].

### Statistical analysis

#### Epidemiological trends analysis

We used a log-linear model for joinpoint regression to analyze trends in the incidence rate of measles infection from 2005 to 2022. The analysis was stratified by age groups: 0-7 months, 8-23 months, 2-4 years, 5-14 years, 15-19 years, 20-59 years, and ≥ 60 years. Joinpoint regression analysis was utilized to calculate the annual percentage changes (APCs) and their corresponding 95% confidence intervals (CIs) for each trend segment. The significance of these changes was assessed using the Z-test (p<0.05) [12]. Statistically significant positive APCs indicate an increasing trend in the incidence rate of measles infection, whereas negative APCs suggest a decreasing trend. When the APCs were not significant (p≥ 0.05), regardless of being positive or negative, the incidence rate was considered stable. The analysis was performed using Joinpoint version 4.8.0.1 [13].

#### Spatial and temporal aggregation analysis

We utilized SatScan version 9.5 to conduct spatiotemporal scan statistics, analyzing the space-time cluster of measles infection in Zhejiang Province [14]. To do this, we created space-time two-dimensional cylinder scanning windows for each city in the province. The size of each window was determined by the city’s share of the total population of Zhejiang Province. We calculated the log-likelihood ratio (LLR) by comparing the actual and theoretical incidence numbers inside and outside these scanning windows. The clusters were ranked according to their LLR values [15], with the highest LLR indicating the primary clustering region, followed by the secondary clustering region, and so on. The relative risk (RR) quantifies the disease risk within the clustering area, representing the likelihood of disease occurrence inside the scanning window compared to outside it. A Monte Carlo simulation was conducted to determine whether the cases were distributed randomly (p<0.05).

#### Bayesian age-period-cohort model

The Bayesian age-period-cohort (BAPC) model has been widely used to analyze the incidence rate or mortality trends of various diseases and to predict future disease burdens. This includes applications in both chronic [16,17] and infectious diseases [18]. Hence, we utilized a BAPC model to forecast the incidence rate of measles infection from 2023 to 2030. This analysis assesses the feasibility of eradicating measles infections by 2030, utilizing an age-period-cohort (APC) model. The model categorizes age groups into 5-year intervals and period groups on an annual basis. Our primary objective was to predict the incidence rate. The identifiability issues of the APC model did not affect the estimation of the incidence rate [19]. To predict the future incidence rate of measles infection, second-order random walks were employed to smooth the prior distributions of age, period, and cohort effects. The principles and formulas of the BAPC model have been thoroughly discussed in prior studies. [18,20]. The analysis used the “INLA” and “BAPC” packages in R version 4.0.5 (R Foundation for Statistical Computing, Vienna, Austria).

### Ethics statement

Collection and analysis of mandatory reported disease surveillance data are considered by the Ethics Review Committee (ERC) of the Zhejiang Provincial Center for Disease Control and Prevention to be routine public health work and does not require specific ERC approval.

## RESULTS

### Epidemiological characteristics of measles infection in Zhejiang Province in 2005-2022

**Figure 1.**
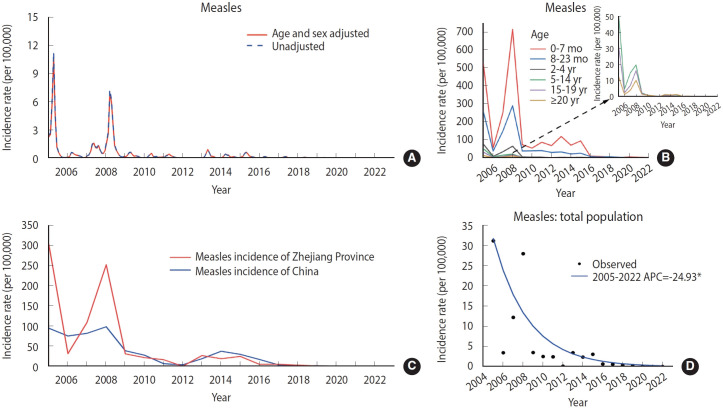
The reported incidence and trends of measles infections in Zhejiang Province from 2005-2022. (A) Age-standardized incidence rate and unadjusted monthly reported incidence rates of measles infections from 2005 to 2022. (B) Annual incidence rates of measles infections in different age groups from 2005 to 2022. (C) The yearly reported incidence rates of measles infection cases in Zhejiang Province and across China from 2005 to 2022. (D) Joinpoint analysis of measles infections from 2005 to 2022. APC, annual percentage change. *p<0.05.

The reported annual ASIR for measles in Zhejiang, China, was 5.24 per 100,000. From 2005 to 2022, there were 7 deaths due to measles in 2005, 1 in 2007, and 4 in 2008; no deaths were reported in the subsequent years. The highest reported ASIR for measles infection occurred in 2005, reaching 31.19 per 100,000 (Figure 1A). In that year, 14,317 confirmed measles cases were reported, and in 2008, there were 12,782 cases. The incidence rates in both years were approximately 300 per 1,000,000, roughly 3 times the national average for those respective years in China. Since 2009, the measles incidence rate in Zhejiang Province has consistently been below the national level (Figure 1A and C). There was also a sustained and significant downward trend in the reported ASIR of measles infection from 2005 to 2022, with an APC of -24.93% (p<0.05). Over this period, the rates of measles decreased by 99.92%. Additionally, the reported ASIR for measles infection fell below 0.1 per 100,000 after 2020 (Figure 1D). The highest annual average incidence rate of measles infection was observed in infants aged 0-7 months, at 119.00 per 100,000 (Figure 1B).

**Figure 2.**
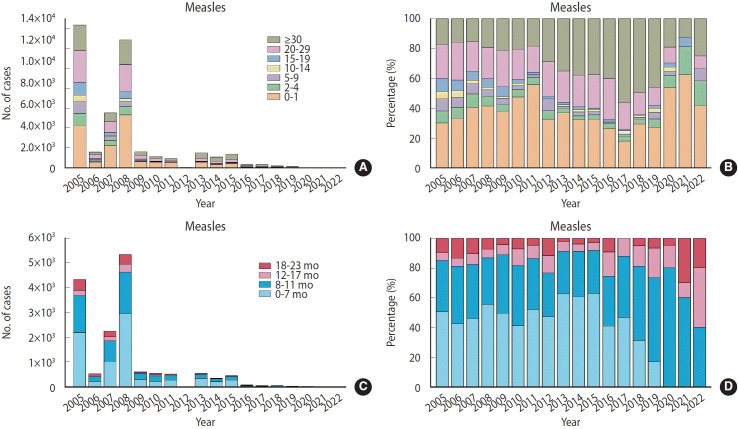
The number of cases (A, C) and percentage (B, D) of measles infections in different age groups from 2005 to 2022.

The proportion of cases in adults over 30 increased steadily until 2017, after which it began to decline (Figure 2A and B). The incidence rate of measles was particularly high among children aged 0-1 year, especially infants aged 0-7 months, and decreased sharply as age increased. From 2005 to 2022, significant numbers of measles cases were consistently reported in children aged 0-1 year, predominantly within the 0-7 months age group. Notably, measles infections ceased after 2019. However, the incidence rate remained relatively high among children aged 8-11 months (Figure 2C and D).

**Figure 3.**
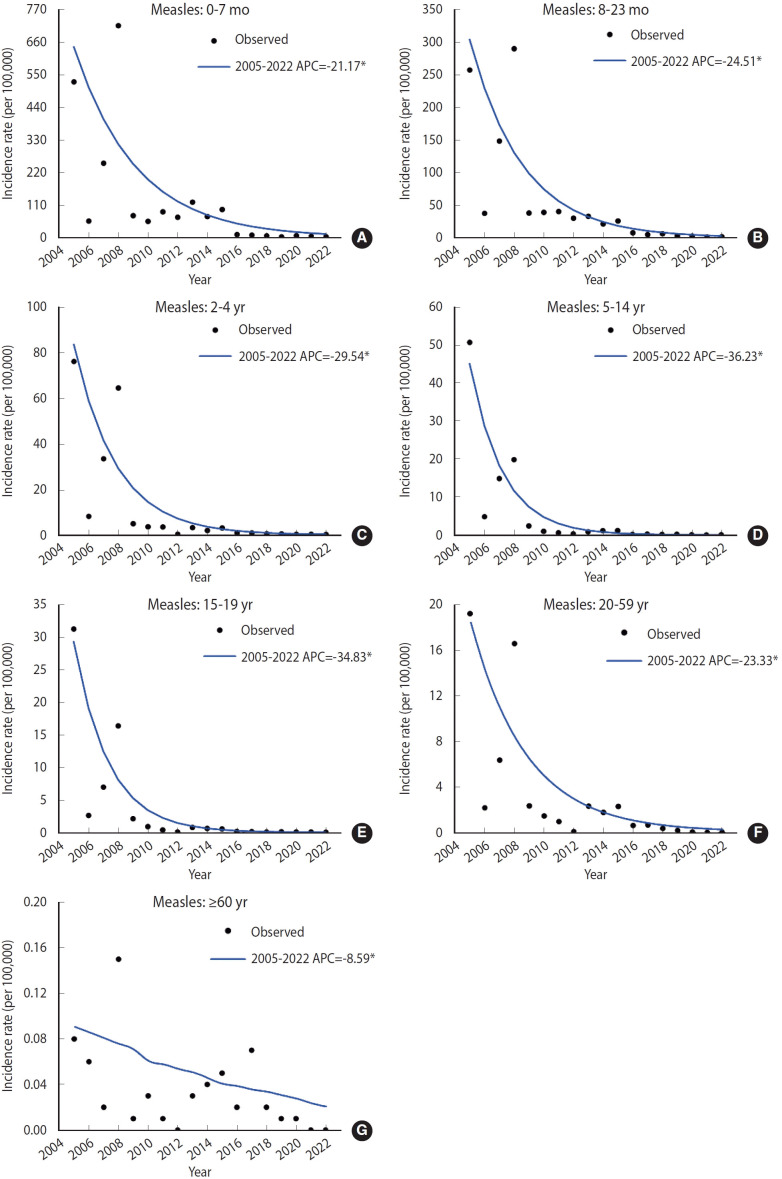
Joinpoint analysis of the incidence rate of measles infections in different age groups (A: 0-7 months, B: 8-23 months, C: 2-4 years, D: 5-14 years, E: 15-19 years, F: 20-59 years, and G: ≥60 years) in Zhejiang Province from 2005 to 2022. APC, annual percentage change. *p<0.05.

The joinpoint analysis revealed a significant and continuous decrease in the incidence rate of measles infection across all age groups (Figure 3A-G).

**Figure 4.**
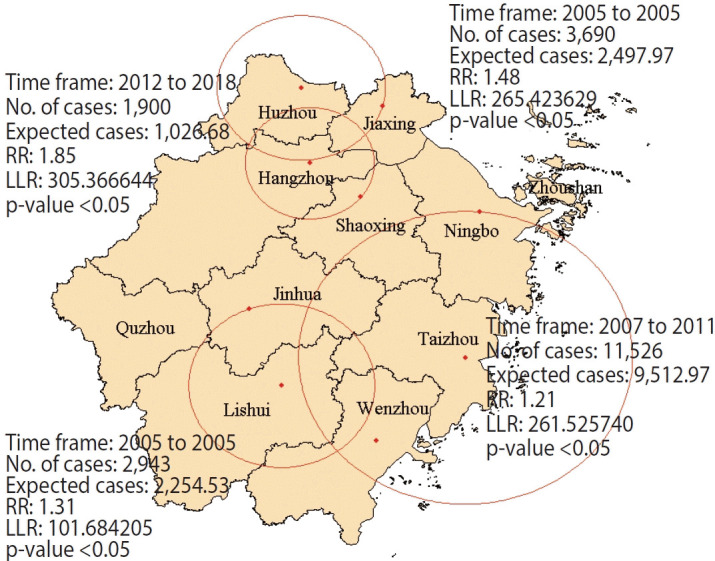
Spatial and temporal aggregation of measles infections in Zhejiang Province from 2005 to 2022. RR, relative risk; LLR, log-likelihood ratio.

The spatial and temporal aggregation analyses identified 4 spatiotemporal aggregation areas for measles infection. The initial aggregation areas were Hangzhou City and Shaoxing City, with the aggregation occurring from 2012 to 2018. During this period, there were 1,900 observed cases compared to 1,026.68 expected cases (RR, 1.85; LLR, 305.37; p<0.05). Secondary spatiotemporal aggregation areas were identified in Huzhou City and Jiaxing City, with the aggregation occurring in 2005. This region reported 3,690 cases against an expected 2,497.97 (RR, 1.48; LLR, 265.42; p < 0.05). The third spatiotemporal aggregation area included Taizhou City, Wenzhou City, and Ningbo City, while the fourth area comprised Lishui City and Jinhua City (Figure 4).

### Prediction of the incidence rate of measles infections in 2023-2030

**Figure 5.**
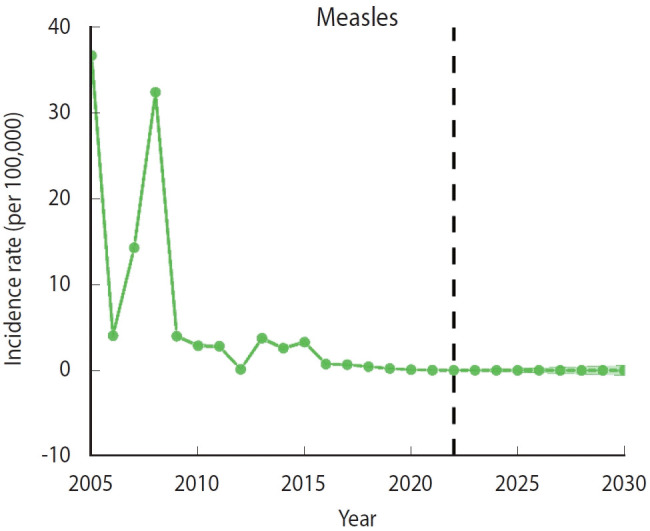
Predicted incidence rates of measles infection for 2023-2030 in Zhejiang Province. The dotted line represents the age-standardized incidence rate, and the shadow represents the 95% credible interval.

The BAPC model predicted a sustained decline in the incidence of measles infections from 2023 to 2030, with a rate that is expected to remain below 0.1/100,000 during this period (Figure 5).

### Measles surveillance: indicators and findings

According to MSS data, the annual rate of discarded measles cases from 2009 to 2014, and the annual rate of discarded measles and rubella cases from 2015 to 2022, exceeded 2 per 100,000, indicating a high sensitivity in measles surveillance. Each year from 2009 to 2022, over 90% of suspected cases were investigated within 48 hours of reporting. The percentage of cases for which a blood specimen was collected increased from 81.94% in 2009 to 99.48% in 2022 (Supplementary Material 1). Additionally, the proportion of laboratory-confirmed measles cases escalated from 39.13% in 2005 to 100% in 2022.

From 2010 to 2022, among all reported measles cases, 41.06% had not received any doses of the measles-containing vaccine (MCV), 9.0% had received 1 dose, 3.1% had received at least 2 doses, and 41.4% had an unknown vaccination status. Between 2005 and 2022, 483 measles virus isolates were obtained for genotyping from 11 cities in Zhejiang Province. All measles isolates were of the H1 genotype, with the exceptions of 1 B3, 21 D8, and 16 vaccine-related viruses (Supplementary Material 2).

### Supplementary immunization activities and vaccination coverage

In 2005, 11 cities strengthened their vaccination efforts with support from local governments. The initial target demographic for these vaccination campaigns was children aged 8 months to 7 years. The campaigns successfully administered approximately 3.93 million doses, vaccinating over 97% of the eligible children. In 2008, Zhejiang Province implemented supplementary immunization activities (SIAs) targeting 3 specific groups: children aged 8 months to 6 years, and first-year college students, who received the MV vaccine. The total doses administered were over 3 million for the younger children and 284 thousand for the college students. Additionally, nearly 587,000 ninth-grade students received the MR vaccine. The focus of the 2009 SIAs shifted to ninth-grade students, high school sophomores and seniors, and first-year college students, with more than 150 million eligible students receiving vaccinations. In 2011, the Zhejiang Provincial Government launched a new vaccination campaign targeting 10th-grade students with the MR vaccine, regardless of their previous immunization status. Since then, the Zhejiang provincial government has conducted two rounds of SIAs annually, in winter and spring. The target age groups for these activities varied from 8 months to 4 years, 7 years, or 15 years, depending on the city, with over 79,000 students vaccinated each year. From 2005 to 2022, the reported coverage rates for the first and second doses of MCV in each city consistently exceeded 95%. Furthermore, from 2011 to 2022, the coverage rate for the third dose of MCV also remained above 95% (Supplementary Material 2).

## DISCUSSION

Our analysis revealed a significant decline in measles cases from 2005 to 2022, indicating substantial progress toward eliminating the disease in Zhejiang Province. Decreases were observed across all age groups, including both the vaccine-targeted children aged 0-14 and the non-targeted age groups, suggesting both direct and indirect protection from measles. Although Zhejiang Province experienced two widespread measles outbreaks in 2005 and 2008 [21], with incidence rates notably higher than the national average at those times, the incidence of measles has consistently declined since 2009. The rates have either fallen below the national average or reached levels indicative of low-level endemicity. Due to an effective and sensitive surveillance system [22], high immunization coverage, and a series of large-scale SIAs [23,24], the measles incidence rate in Zhejiang Province has steadily decreased since 2015. Based on this series of measures, the COVID-19 pandemic’s onset led to an acceleration in measles prevention and control in Zhejiang Province through NPIs [25]. As a result, from 2021 to 2022, the reported annual incidence rate consistently remained below one case per million population. We are optimistic that Zhejiang is on the path to interrupting measles virus circulation and successfully achieving the goal of eliminating measles.

Infants aged 0-7 months have consistently shown the highest annual incidence rate of measles infection since 2005. However, as the overall number of measles cases has declined, the proportion of cases among adults has increased. This indicates a shift in the age distribution of susceptibility to measles in Zhejiang Province, moving from young children to adults. Li et al. [26] observed that declining birth rates, reduced measles prevalence, and improved vaccination coverage might be driving changes in the age distribution of measles across China. The primary reason for the high measles incidence among children aged 0-7 months is the decrease in maternal antibody levels [1]. Therefore, the first dose of MCV is administered at 8 months of age in China, which is the earliest age for this vaccination globally [27]. The introduction of MCV vaccination for ninth-grade students in Zhejiang Province has led to a significant reduction in measles cases among individuals aged 10-19 years [28]. Despite this, the proportion of adult cases has continued to rise. Adjustments in immunization strategies involving measles-containing vaccines have altered the previous patterns of the measles epidemic. However, the increasing number of measles cases in adults presents significant challenges to achieving the goal of measles elimination [3].

Our study revealed that over 80% of measles cases involved individuals with no history of immunization or unknown immunization status. This finding highlights the preventive effectiveness of the MCV against measles. Historically, measles vaccination has been widely accepted by the Chinese public, with virtually no opposition from anti-MV groups. However, during the 2009 Guangdong provincial SIA, public concerns emerged regarding the necessity of administering a third vaccine dose to children who had already received two documented doses [29]. In early 2010, there were both unsubstantiated and substantiated reports of adverse events following immunization after vaccination with various antigens [30]. With the recent decline in measles cases, there has been a decrease in vigilance about the disease. These developments have increased public concerns about vaccines in general. The long-term impact on vaccine uptake remains to be determined.

Monitoring progress toward the measles elimination goal requires a measles surveillance system that is both sensitive and specific. The case-based measles surveillance system in Zhejiang Province is now supported by high-quality laboratory services. The H1 genotype, which had been endemic in China for over 25 years [31], was also a predominant strain in Zhejiang Province until its last detection in 2017. This surveillance system has improved our understanding of the genetic diversity of measles viruses in the region. In recent years, Zhejiang Province has reported only a few dozen measles cases annually. We have conducted thorough investigations into potential sources of infection, including imported cases from other countries or regions. For each case, we recorded the patient’s recent travel history and any interactions with individuals exhibiting fever and rash symptoms. After collecting and testing throat swab and blood samples from measles patients, we isolated and cultured the measles virus. Virological surveillance plays a vital role in identifying circulating viruses, tracking the importation of new viruses, and monitoring the disappearance of specific wild-type MV lineages [32].

Using incidence data from 2005 to 2022 for all 11 cities in Zhejiang Province, we demonstrate that the municipal burden of measles varied significantly, with some cities being highly connected and experiencing synchronous outbreaks. Our study identified Hangzhou and Shaoxing as first-level spatiotemporal aggregation areas for measles. However, neither Quzhou nor Zhoushan fell within any of the spatiotemporal clusters identified through mapping measles cases during this period. As the capital of Zhejiang Province, Hangzhou boasts a population exceeding 10 million. In contrast, Quzhou and Zhoushan, characterized by their mountainous and island landscapes, have a combined population of less than a quarter of that of Hangzhou. Factors such as population density and high mobility significantly influence the spread of respiratory diseases such as measles [33,34]. The first spatiotemporal aggregation areas were particularly evident between 2012 and 2018, a period when measles incidence was at its lowest in Zhejiang Province. This pattern suggests that, although the total number of cases was low, the burden of measles infection in these regions was much higher compared to the rest of Zhejiang. These areas likely acted as refuges for measles when transmission was interrupted elsewhere [35]. Our study also observed a positive correlation between temporal clustering and years of high incidence.

Measles seroepidemiological studies are crucial in supporting efforts to eliminate the disease. In 2020, a study in Zhejiang Province involved 2,740 individuals ranging in age from 0 years to 59 years revealed a measles antibody prevalence of 85.30% [36]. This prevalence rate is still some distance away from the WHO’s minimum threshold for measles eradication, which requires a sustained population immunity level of at least 95% [1].

The model predicts that measles incidence in Zhejiang Province will remain below 1 per million from 2023 to 2030. However, measles serves as a barometer of the quality of the immunization program. This highly contagious disease is prone to resurgence, even in regions that have achieved measles elimination [7]. From 2020 to 2022, measures implemented by the Chinese government to control the COVID-19 pandemic—including temporary lockdowns, mask-wearing, social distancing, improved personal hygiene, and restricted travel [37]—may have been effective in preventing the spread of infectious respiratory diseases such as measles. In the post-COVID-19 era, to sustain the low incidence rate of measles, rigorous enforcement of all routine measles control measures must be maintained without any relaxation. These measures include achieving high immunization coverage, implementing sensitive surveillance systems, and ensuring thorough investigation and management of each confirmed case, as well as tracing the source of infection.

This study has several limitations related to its reliance on case reporting. First, the data are dependent on cases recorded by the national surveillance system, and potential underreporting—possibly due to less severe symptoms—may bias the results. Second, it is crucial to consider previous seroepidemiological studies when analyzing the situation for measles elimination. However, this study only references the results from a seroepidemiological survey conducted in Zhejiang Province in 2018. This limitation prevents us from depicting the changes in measles seroprevalence among the residents of Zhejiang Province from 2005 to 2022. Therefore, further research is necessary to comprehensively assess the effectiveness of measles prevention and control efforts.

## Figures and Tables

**Figure f6-epih-46-e2024075:**
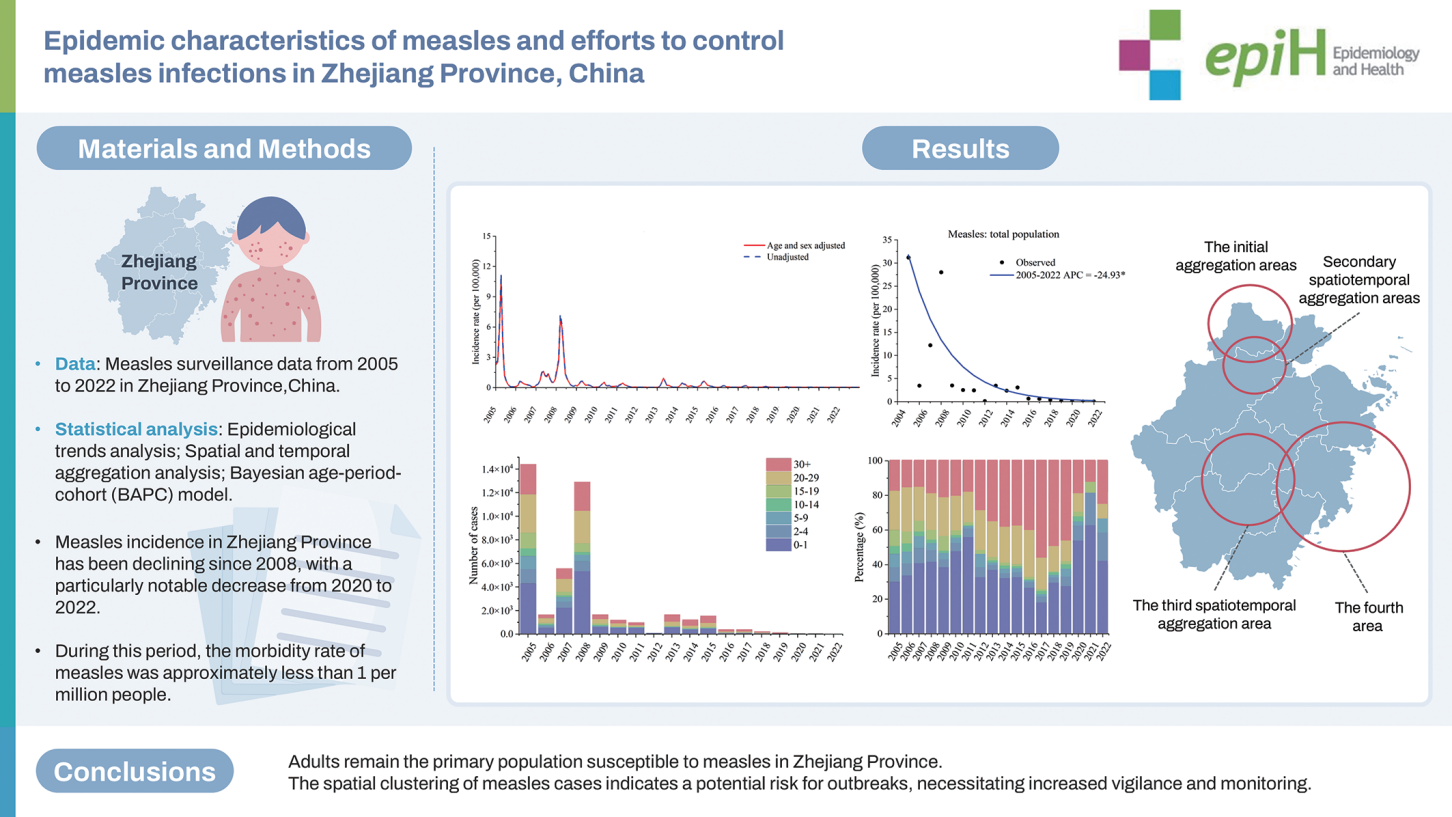


## Supplementary materials

Supplementary Material 1. Table The Performance of Measles Surveillance Information Reporting and Management System in Zhejiang Province from 2009–2022

Supplementary Material 2. Table Reported cases of measles, by outbreaks, isolate genotypes, and vaccination status, and surveillance indicators -Zhejiang, 2005–2022
