# Supplementary material for: Epidemic characteristics of measles and efforts to control measles infections in Zhejiang Province, China
Source: Epidemiol Health. 2024 Sep 11;46:e2024075. doi: 10.4178/epih.e2024075 (PMC11826004; doi:10.4178/epih.e2024075)
Supplement: Supplementary Material 1. — Table The Performance of Measles Surveillance Information Reporting and Management System in Zhejiang Province from 2009–2022 [file epih-46-e2024075-Supplementary-1.docx]

**Supplementary Material 1:**

**Table The Performance of Measles Surveillance Information Reporting and Management System in Zhejiang Province from 2009–2022**

| **Monitoring indicators*** | **2009** | **2010** | **2011** | **2012** | **2013** | **2014** | **2015** | **2016** | **2017** | **2018** | **2019** | **2020** | **2021** | **2022** |
| --- | --- | --- | --- | --- | --- | --- | --- | --- | --- | --- | --- | --- | --- | --- |
| **Indicators of Sensitivity** |  |  |  |  |  |  |  |  |  |  |  |  |  |  |
| No. of discarded cases per 100,000, national level (target ≥ 2)** | 2.98 | 2.48 | 4.66 | 4.19 | 3.03 | 2.66 | 2.94 | 2.37 | 2.45 | 2.31 | 2.63 | 2.29 | 2.19 | 2.30 |
| **Indicators of Timeliness** |  |  |  |  |  |  |  |  |  |  |  |  |  |  |
| % of suspected cases adequately investigated  ≤ 48 h after notification (target ≥ 80) | 90.36 | 95.42 | 98.53 | 99.91 | 99.81 | 99.57 | 99.33 | 99.31 | 99.66 | 99.38 | 98.81 | 99.03 | 99.11 | 99.03 |
| % of blood specimens reported IgM results within 7 d (target ≥ 80)*** | 89.12 | 94.81 | 95.38 | 99.42 | 98.79 | 98.52 | NA | NA | NA | NA | NA | NA | NA | NA |
| % of blood specimen deliveried within 3 d (target ≥ 80) | NA | NA | NA | NA | NA | NA | 99.26 | 98.79 | 99.19 | 99.1 | 97.33 | 99.39 | 97.84 | 99.42 |
| % of samples tested ≤ 4 d after specimen receipt in laboratory (target ≥80) | NA | NA | NA | NA | NA | NA | 97.2 | 97.69 | 96.71 | 97.25 | 96.83 | 96.6 | 99.05 | 98.12 |
| **Indicators of Specificity** |  |  |  |  |  |  |  |  |  |  |  |  |  |  |
| % of Cases within blood specimen (target ≥ 80) | 81.94 | 87.47 | 93.21 | 99.69 | 99.61 | 99.09 | 99.14 | 99.14 | 99.44 | 99.44 | 99.32 | 99.03 | 99.04 | 99.48 |
| % of outbreaks with blood confirmation (target ≥ 80) | 100.00 | 100.00 | 100.00 | NA | 100.00 | 100.00 | 100.00 | 100.00 | 100.00 | 100.00 | 100.00 | NA | NA | NA |
| % of outbreaks with viral specimen collected(target ≥ 80) | 75.00 | 83.33 | 91.67 | NA | 94.44 | 100.00 | 100.00 | 100.00 | 100.00 | 100.00 | 100.00 | NA | NA | NA |

*The data source was from the Measles Surveillance System. From 2009–2014, sensitivity, timeliness, and specificity were for measles only, but since 2015, all indicators have been calculated according to measles and rubella.

**From 2009–2014, the system calculated discarded measles cases as the numerator of the rate of discarded cases. Since 2015, the numerator has discarded measles and rubella cases.

***The timeliness indicator of blood specimen reported IgM results within 7 d was changed into two indicators of blood specimen delivered within 3 d and samples tested within 4 d after specimen receipt in the laboratory since the National Programme of Measles Surveillance was revised in 2014.

**Table 2. Reported cases of measles, by outbreaks, isolate genotypes, and vaccination status, and surveillance indicators -Zhejiang, 2005–2022**

| **Characteristic** | **2005** | **2006** | **2007** | **2008** | **2009** | **2010** | **2011** | **2012** | **2013** | **2014** | **2015** | **2016** | **2017** | **2018** | **2019** | **2020** | **2021** | **2022** |
| --- | --- | --- | --- | --- | --- | --- | --- | --- | --- | --- | --- | --- | --- | --- | --- | --- | --- | --- |
| Measles incidence, cases per million population | 303.42 | 32.05 | 109.40 | 252.61 | 31.40 | 22.16 | 17.18 | 0.95 | 27.24 | 19.63 | 25.16 | 5.89 | 5.80 | 3.48 | 1.88 | 0.63 | 0.25 | 0.18 |
| No. of measles cases | 14317 | 1570 | 5448 | 12728 | 1609 | 1148 | 935 | 52 | 1492 | 1079 | 1386 | 326 | 324 | 197 | 108 | 37 | 16 | 12 |
| No. of Laboratory-confirmed measles cases (%)* | 39.13 | 44.97 | 49.45 | 70.88 | 70.04 | 85.19 | 99.47 | 100.00 | 99.87 | 99.81 | 99.71 | 99.69 | 99.69 | 98.98 | 100.00 | 97.30 | 93.75 | 100.00 |
| No. of measles-related deaths | 7 | 0 | 1 | 4 | 0 | 0 | 0 | 0 | 0 | 0 | 0 | 0 | 0 | 0 | 0 | 0 | 0 | 0 |
| Measles virus genotypes (no. of identified) | H1 (7) | H1 (2) | H1 (4) | H1 (6) | H1 (2) | H1 (6) | H1 (12); A(7) | H1 (9) | H1 (69) | H1 (2); B3 (1); A (1) | H1 (256) | H1 (6) | H1 (64) ; D8 (5); A(2) | NA | D8 (16); A (3) | NA | A (3) | NA |
| No. of outbreaks reported§ | 21 | 2 | 8 | 14 | 9 | 12 | 12 | 0 | 18 | 19 | 32 | 12 | 7 | 7 | 1 | 0 | 0 | 0 |
| No. of outbreak-related cases | 426 | 8 | 56 | 89 | 27 | 53 | 51 | 0 | 52 | 71 | 107 | 28 | 28 | 17 | 6 | 0 | 0 | 0 |
| Median no. of cases per outbreak (range) | 13(3–121) | 4(4–4) | 7(3–15) | 4(3–14) | 2(2–6) | 4(2–10) | 3(2–14) | 0 | 2(2–14) | 3(2–8) | 2(2–16) | 3(2–13) | 4(2–10) | 2(2–7) | 6 | 0 | 0 | 0 |
| Median outbreak duration, days (range) | 34(21–120) | 31(29–33) | 35(28–46) | 29(23–40) | 25(22–30) | 27(23–35) | 35(26–49) | 0 | 29.5(23–43) | 30(21–40) | 29(21–48) | 28(21–37) | 26(21–45) | 29(21–48) | 23 | 0 | 0 | 0 |
| No. of vaccine doses received by measles patients† | | | | | | | | | | | | | | | | | | |
| 0 | NA | NA | NA | NA | NA | 538 | 546 | 23 | 658 | 422 | 529 | 79 | 71 | 38 | 16 | 1 | 0 | 0 |
| 1 | NA | NA | NA | NA | NA | 128 | 84 | 6 | 118 | 78 | 83 | 25 | 30 | 41 | 16 | 18 | 8 | 5 |
| ≥ 2 | NA | NA | NA | NA | NA | 41 | 25 | 1 | 31 | 19 | 38 | 14 | 18 | 8 | 13 | 5 | 6 | 3 |
| Unknown | NA | NA | NA | NA | NA | 441 | 282 | 22 | 685 | 560 | 736 | 208 | 205 | 110 | 63 | 13 | 2 | 4 |
| Administrative MCV1 coverage (%)** | 98.99 | 97.74 | 99.95 | 99.98 | 99.98 | 99.57 | 99.80 | 99.81 | 99.86 | 99.83 | 99.86 | 99.81 | 99.67 | 99.71 | 99.66 | 99.65 | 99.74 | 99.72 |
| Administrative MCV2 coverage (%)** | 97.63 | 98.32 | 99.93 | 99.94 | 97.89 | 99.32 | 99.76 | 99.94 | 99.81 | 99.79 | 99.75 | 99.80 | 99.44 | 99.63 | 99.59 | 99.57 | 99.74 | 99.68 |
| Administrative MCV3 coverage (%)** | NA | NA | NA | NA | NA | NA | 98.66 | 95.67 | 95.52 | 98.43 | 98.34 | 98.27 | 98.45 | 98.50 | 98.62 | 98.88 | 98.98 | 99.15 |
| No. of persons vaccinated in SIAs§§ | 3933107 | NA | 334100 | 4301680 | 1506804 | 2421830 | 518277 | 169105 | 239097 | 158986 | 146339 | 97085 | 98388 | 107096 | 91701 | 110978 | 88265 | 79112 |

Abbreviations: MCV = measles-containing vaccine; MCV2 = second dose of MCV; NA = unavailable; SIA = supplementary immunization activity.

* Defined as a case that meets the suspected case definition and is laboratory-confirmed (serologically or virologically) as measles.

§ In China, from 2005–2009, a measles outbreak was defined as the occurrence, within 21 d, of three or more confirmed measles cases in a village, district, school, or similar unit or five or more confirmed measles cases in a township, according to the national Measles Surveillance Program which was issued in 2003. After the new version of the National Measles Surveillance Program was revised in 2009, a measles outbreak was defined as the occurrence, within 10 d, of two or more confirmed measles cases in a village, district, school, or similar unit or five or more confirmed measles cases in a township, since 2010.

† No. of doses of MCV received by the patient as of the onset of measles illness.

** Coverage Monitoring: Each city reports the administrative coverage of the first and second doses of measles-containing vaccine (MCV1 and MCV2) utilizing denominators based on the number of children registered as ‘‘target’’ in the routine EPI system. Each city also reports vaccination data for 9th-grade students as the third dose of MCV. This includes the number of adolescents eligible for vaccination, the actual number of vaccinated, and the primary reasons for not vaccinating.

§§ Supplementary Immunization Activities (SIAs): SIAs were mass immunization campaigns to vaccinate all children in specified age groups. To calculate SIA coverage, the Centers for Disease Control in each city categorized the number of vaccinated children by the total number of children targeted for vaccination by county during SIAs. Upon completion of the SIA, aggregated coverage data were transmitted to the provincial level.
